# Metabolic pathways associated with cardiometabolic risk effects on cognition in middle-aged adults: the CARDIA study

**DOI:** 10.1007/s11306-026-02458-w

**Published:** 2026-06-17

**Authors:** Sujin Kang, Timothy Ebbels, Queenie Chan, Luke Whiley, Elaine Holmes, Kristine Yaffe, Katie Meyer, Julian Griffin

**Affiliations:** 1 Department of Metabolism, Diabetes and Reproduction, Imperial College London, London, UK; 2 Ageing Epidemiology Research Unit, School of Public Health, Imperial College London, London, UK; 3 Department of Epidemiology and Biostatistics, School of Public Health, Imperial College London, London, UK; 4 School of Diagnostic and Therapeutic Sciences, Curtin University, Perth, WA Australia; 5 Centre for Computational and Systems Medicine, Murdoch University, Perth, WA Australia; 6 Institute for Neurosciences, University of California, San Francisco, CA USA; 7 Department of Nutrition, University of North Carolina, Chapel Hill, NC USA; 8 The Rowett Institute, University of Aberdeen, Aberdeen, UK

**Keywords:** Cardiometabolic risk factors, Cognitive impairment, Metabolites, MRI markers, Structural equation modelling

## Abstract

**Background:**

Evidence increasingly suggests a connection between cardiovascular disease and brain health in later life; however, the mechanistic pathways from human studies remain unclear. This study aimed to investigate whether urinary metabolites account for part of the association between cognition and cardiometabolic risk.

**Methods:**

Data from 606 participants (aged 48–60; 55% female; 45.5% Black/African American) in the Year 30 follow-up of the Coronary Artery Risk Development in Young Adults Study were analyzed. Urinary metabolites were profiled using nuclear magnetic resonance spectroscopy and liquid chromatography-mass spectrometry; brain magnetic resonance imaging data were available for 281 participants. Structural equation models were used to assess pathways linking cardiometabolic factors to cognitive outcome, with urinary metabolites and brain MRI-derived parameters as mediators.

**Results:**

Fasting glucose showed a negative association with cognition. Valine, isoleucine, leucine, and phenylalanine were positively associated with fasting glucose. Valine and aminoadipic acid also showed positive associations between fasting glucose and cognition, while tryptophan was correlated with both fasting glucose and cognition. Indole-3-acetic acid showed negative associations with systolic blood pressure and fasting glucose. Brain MRI-derived parameters in memory-related medial temporal areas were associated with waist circumference.

**Conclusions:**

Urinary metabolites and brain imaging markers were linked with hyperglycemia, obesity, and cognitive performance, highlighting multimodal biomarkers relevant to global cognitive function in individuals with cardiometabolic risk.

**Supplementary Information:**

The online version contains supplementary material available at https://doi.org/10.1007/s11306-026-02458-w.

## Introduction

Hypertension, dyslipidaemia, hyperglycemia, and obesity play significant roles in cognitive health. For instance, hypertension is linked to brain damage and an increased dementia risk, with studies showing associations with cognitive neurodegenerative disorders and vascular dementia (VaD) (Abell et al., 2018; Poblador-Plou et al., 2014; Yano et al., 2014); dyslipidaemia, particularly high total cholesterol in midlife, has also been associated with Alzheimer’s disease and cognitive decline (Anstey et al., 2017; Kivipelto et al., 2001; Mielke et al., 2010; Notkola et al., 1998); hyperglycemia, which is prevalent in patients with diabetes, is correlated with cognitive impairment (Cox et al., 2005; Sommerfield et al., 2004); and obesity contributes to cognitive decline and dementia risk (Anjum et al., 2018; Dye et al., 2017; Whitmer et al., 2005). A healthier lifestyle, including healthy diet and physical activity, supports cognitive function and can build cognitive reserve for later life (Clare et al., 2017).

Although the association between metabolic diseases, such as type 2 diabetes and obesity, and cognitive health has been investigated, the underlying mechanisms by which these conditions impair cognitive performance remain unclear. Similarly, while the connection between vascular health and cognitive decline in later life has been studied, there is a requirement for greater emphasis on the association between vascular health and cognitive decline in middle-aged adults (Alonso, et al., 2017; Grodstein, 2007). A better understanding of these underlying physiological processes and how the metabolic syndrome contributes to cognitive decline could have a major impact on public health.

This study aimed to elucidate the link between metabolic and cardiovascular health and cognition by exploring how metabolites account for this relationship. This investigation used data from the Coronary Artery Risk Development in Young Adults (CARDIA) Study, measured at Year 30 when subjects were aged 48–60 years. The CARDIA Study provided an opportunity to examine associations between exposures and outcomes in this U.S. cohort, while accounting for potential confounding factors and other exposures that might influence the relationship between cardiovascular health and cognition. Our study hypothesizes that, in the presence of cardiometabolic risk, urinary metabolites are associated with cognition outcomes in the study population.

The primary aim of this study was to investigate whether urinary metabolites, measured by targeted metabolomics using a predefined panel relevant to systemic metabolism, alongside brain MRI-derived parameters, serve as prognostic markers of cognitive performance in relation to cardiometabolic risk factors. Furthermore, significant exposures and potential confounding factors among clinical, lifestyle, and sociodemographic factors were examined (Kang, 2021).

## Methods

### Study population

This study focuses on the CARDIA sub-cohort, consisting of 606 participants aged 48–60 years who completed cognitive tests at Year 30 follow-up examination (2015–16). High-resolution proton nuclear magnetic resonance (^1^H NMR) spectroscopy and the liquid-chromatography mass spectrometry (LC–MS) of urine were available for 606 participants, courtesy of the National Phenome Center, Imperial College London (https://phenomecentre.org/). Brain MRI data for 281 participants at Year 30 were obtained through collaboration with the University of Alabama at Birmingham, Northwestern University, University of Minnesota, and Kaiser Foundation Research Institute (documented at https://sites.uab.edu/cardia/for-researchers/exam-materials/data-collection-forms/data-collection-forms-year-30/). Only participants with quality control (QC)-passed data for all MRI variables were included, resulting in a smaller sample size, particularly for CBF measures.

### Cardiometabolic risk factors and other exposures

In the analysis, the cardiometabolic risk factors included hypertension (systolic blood pressure (SBP), diastolic blood pressure (DBP)), dyslipidaemia (high-density lipoprotein cholesterol, low-density lipoprotein cholesterol, triglycerides (mg/dl)), hyperglycemia (fasting glucose (mg/dl), HOMA-equation IR, eGFR, and insulin), and (abdominal) obesity (body mass index (BMI) and waist circumference (WC)). In addition, the 10-year Framingham risk was used as a cardiovascular disease (CVD) risk indicator (Supplementary Table 1). Although the Framingham score incorporates some of the individual cardiometabolic factors, it provides a validated estimate of 10-year CVD risk, capturing the combined effect and complementing the analysis of individual risk factors. Lifestyle exposures included physical activity, a summary diet score, smoking, and alcohol consumption. Sociodemographic exposures encompassed variables such as sex, age, race, center, education up to Year 30, and family income. Health-related information, including depression score, medical/family history of dementia, sleep quality, medication status (hypertension, hypercholesterol, and depression therapies), and serum and urinary creatinine (mg/dL), were examined (Gooding et al., 2017; Parker et al., 2007; Pereira et al., 1997; Sajatovic & Ramirez, 2012).

### Urinary metabolites measured by ^1^H NMR and LC–MS

A total of 47 metabolites and 28 amino acids were measured at Year 30 using NMR spectroscopy (mmol/L) and a targeted LC–MS panel (µM), respectively, selected for their established relevance to systemic metabolism. Normalization was performed using the probabilistic quotient normalization (PQN) method, with high-resolution one-dimensional (1D) NMR spectroscopy data used to compute the normalization factor for each individual’s metabolite profile. Urine samples collected during 2010–11 were subjected to NMR spectroscopy using a Bruker Sample Track system. The supernatant and urine buffer were transferred into a SampleJet NMR tube following centrifugation, resulting in a final sodium azide concentration of 0.2 mM (Dona et al., 2014). In addition, LC–MS methods were used for the analysis of amino acids and metabolites in the tryptophan pathway in urine (Lewis et al., 2016). The precision of the retention times in the chromatographic methods was evaluated using reference standards and internal QC samples (Lewis et al., 2016). Data correction techniques, including the automatic control of the MS detector voltage and feature-specific LC–MS data correction procedures such as curve fitting and LOESS regression, were applied to minimize technical variation (Dunn et al., 2011; Kloet et al., 2009; Lewis et al., 2016). No discernible batch effects were observed during the exploratory data analysis. Subsequent analyses conducted verification of urinary metabolite data, as well as examination of missing metabolites and determination of cases for analysis. Selected urinary metabolites were identified in a data-driven manner from the full set of measured metabolites.

### Brain MRI-derived parameters

Measurements of total intracranial volume, brain tissue volume, gray and white matter (WM) volume in cubic centimeters (cc^3^), and cerebrospinal fluid volumes (cc^3^) were investigated in terms of total and abnormal tissue volumes (cc^3^), cerebral blood flow (mm^3^/100gms/min), and fractional anisotropy (FA). Measurements of brain structures associated with the successive stages of memory processing, such as the hippocampus, medial frontal cortex, and anterior cingulate gyrus, which play roles in encoding, storage, and retrieval, were investigated (Bubb et al., 2017; Cerf-Ducastel & Murphy, 2006; Melton, 1963). Furthermore, the temporal lobe volumes and entorhinal area were investigated. Wide interindividual and sex-dependent variations in head size were observed. In the structural equation modeling (SEM) was used to perform mediation analyses. Intracranial volume (ICV) was included as a covariate to account for individual differences in head size that may account for the relationship between waist circumference (WC) and intracranial tissue volulme (ICV), allowing partitioning of variance, clarifying structural relationships among variables, and examining sex-dependent differences in head size (Sanchis-Segura et al., 2020).

### Outcome measure: adjusted composite cognitive score

The study used the adjusted composite cognitive score to investigate midlife brain health, with scores below 0 indicating impaired cognition. The composite cognitive measures were employed in the analysis with the objective of reducing floor and ceiling artifacts and other forms of measurement error (Tuokko & Hultsch, 2019). The measures were based on the following tests: the Rey Auditory Verbal Learning test (RAVLT), the Digit Symbol Substitution test (DSST), the reversed Stroop test score, and the Montreal Cognitive Assessment (MoCA). The MoCA is a brief cognitive screening tool known for its high sensitivity and specificity in detecting mild cognitive impairment (MCI) using a threshold of < 26 (Nasreddine et al., 2005). The adjusted composite cognitive score, which included the reversed Stroop test, was calculated as the sum of each z-score for these four tests, with a mean of 0.024 and a standard deviation of 3.18.

### Pathway analysis of urinary metabolites and brain MRI-derived parameters

**Fig. 1:**
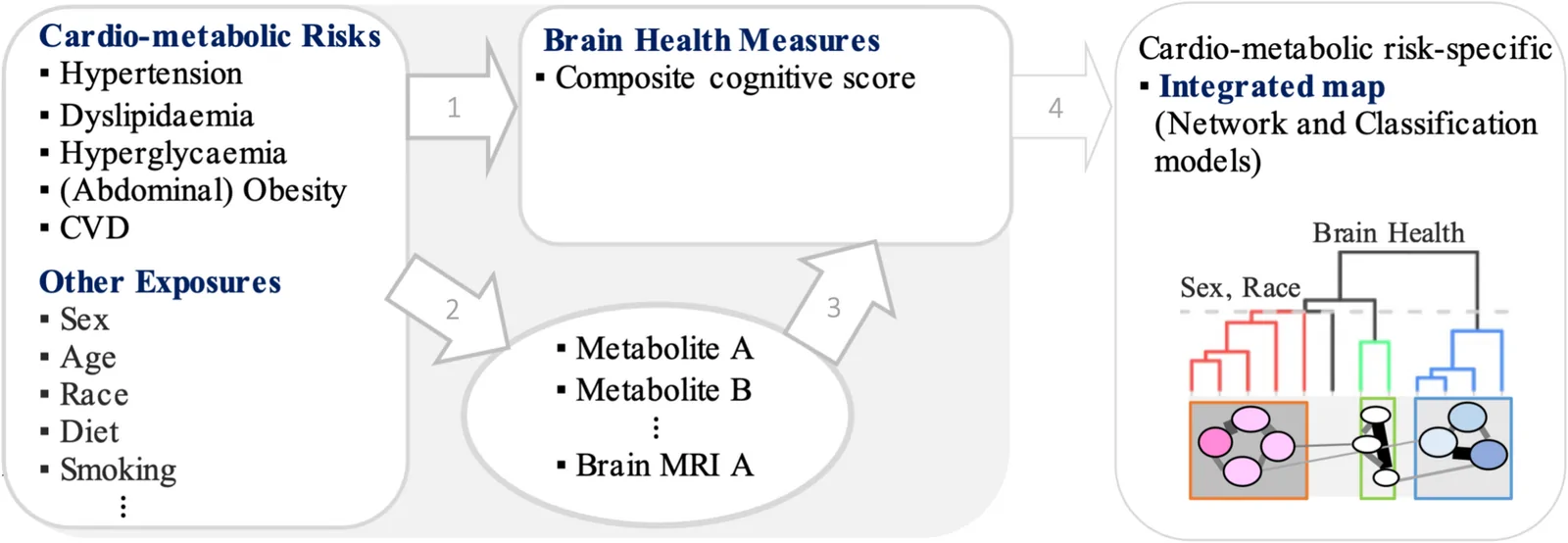
Overview of study objectives and analytical plan.[1–3] The pathway framework of Baron & Kenny (1986) comprises three sets of regression: X → Y, X → M, and X + M → Y.[4]A cardio-metabolic risk-specific integrated map of cognition.

First, analyses were conducted to investigate whether urinary metabolites (M) account for part of the assocation between adjusted composite cognition (Y) and each cardiometabolic risk factor (X), i.e., DBP, fasting glucose, HOMA-equation IR, fasting insulin, BMI, WC, and the 10-year Framingham Risk (Fig. 1 [1–3]) (Baron & Kenny, 1986).

In each SEM, significant cardiometabolic risk factors and covariates (age, sex, race, education, CES-D, smoking status, antihypertensive medication use, depression medication, sleep quality, and family income, as appropriate) were included as exogenous variables, with metabolites as mediators and cognition as the outcome. Direct and indirect effects were estimated based on the regression framework (X → Y, X → M, and X + M → Y). To reduce model complexity and mitigate overfitting, an ensemble model strategy was adopted; each SEM model per cardiometabolic risk factor was conducted, and based on statistical significance, a list of metabolites was selected to predict the overall outcome (Supplementary Table 2).

**Fig. 2:**
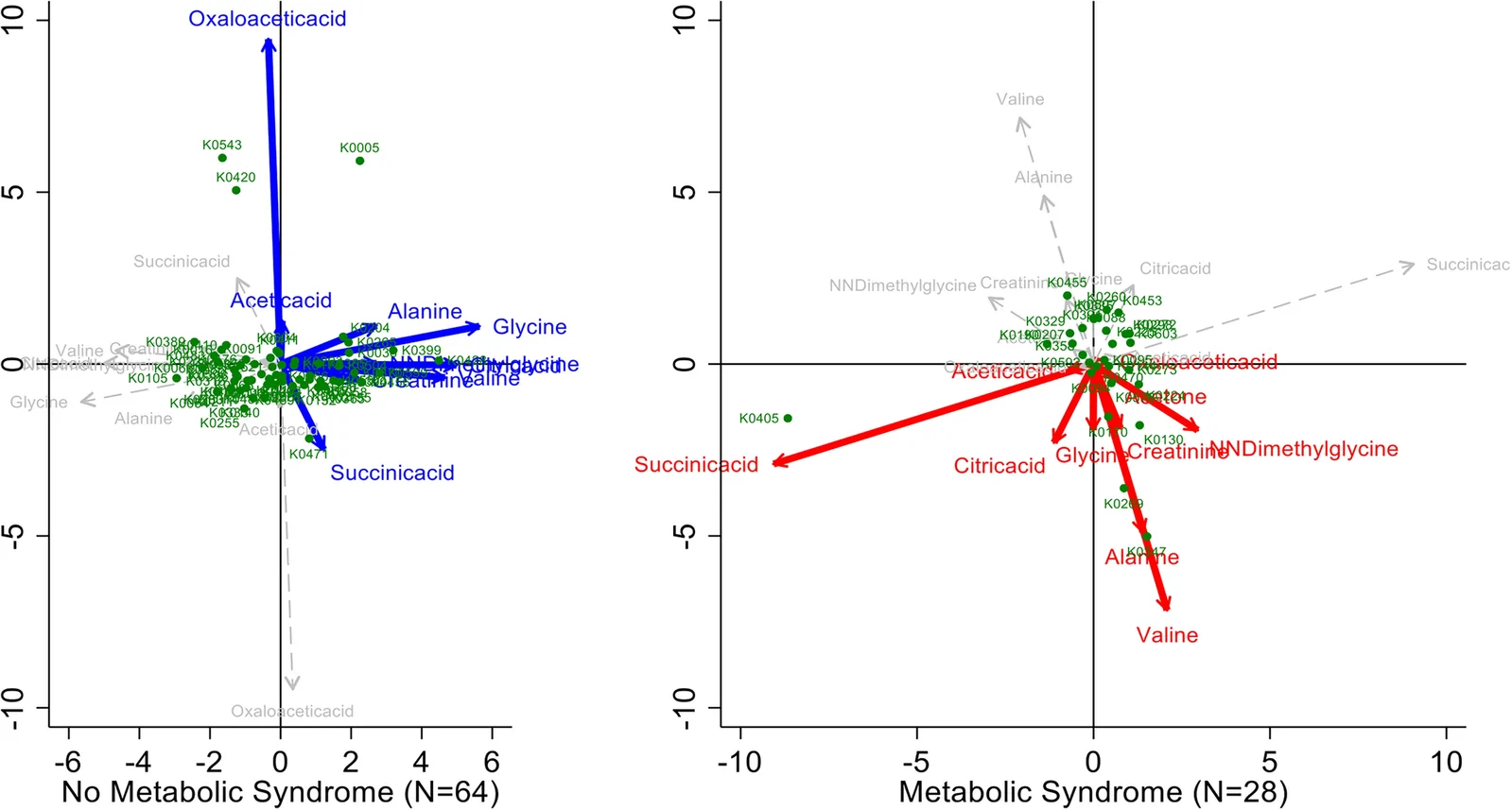
The biplot for (1) no metabolic syndrome and (2) metabolic syndrome (N = 64 vs. 28) groups using PCA for the PQN-normalized NMR data; the gray arrows are a reflection of negative directions

Furthermore, models based on cross-validation for the metabolites were assessed as another method to address potential issues. This involved calculating the variances and residual variance using the predicted values obtained through cross-validation, which provides a certain level of control for potential overfitting during the training process (Jia, 2017). Once each model per cardiometabolic risk factor had been constructed, “significant” (*p* < 0.05) cardiometabolic factors, exposures, intermediate variables, and confounding factors were included in one final non-recursive structural equation model with correlated errors. The SEMs with measurement errors in metabolites from NMR or LC–MS were conducted (Fig. 2). This approach refers to the base estimators from each model per cardiometabolic risk factor, and it was chosen to maintain statistical power over other model fitting methods. Furthermore, a cardiometabolic risk factor “per category” was included in the final model, with a preference for including more intermediate variables in each association.

The models used standardized metabolite scales (i.e., z-score metabolites) to provide additional benefits, including demonstrating good multicollinearity, a good log-likelihood, and the ability to include variables from different units into the final model. A total of 500 bootstrap replications standard errors and confidence intervals were obtained using the ***vce(boostrap)*** option in Stata/IC 16.0 (StataCorp LLC). The convergence of the maximum likelihood estimators in the SEMs was evaluated. To address overidentification, specifically Type I error in small samples, the Swan correction designed for models with several indicators was applied, providing a suite of corrections to the chi-square overidentification test, i.e., the likelihood ratio test of fit, for SEMs. Given the exploratory nature of the analyses and the use of a predefined metabolite panel, formal multiple testing correction was not applied. The analyses were conducted for each metabolite measured by NMR spectroscopy and LC–MS, including amino acids and tryptophan.

Next, pathway analyses using brain MRI data were conducted. Existing literature supports connections between vascular risk factors, several brain MRI-derived parameters such as brain white/gray matter lesions or volume loss, and cognitive impairment or dementia. Moreover, the SEM was conducted to examine how brain MRI-derived parameters serve as markers for adjusted composite cognition in relation to each cardiometabolic factor at Year 30. All significant associations between each brain MRI-derived parameter and cardiovascular risk factor were included in the total brain MRI models, along with significant associations for urinary metabolites. In addition, classification tree models were applied to identify interpretable patterns linking metabolic phenotypes to cognitive impairment in midlife. For the detailed multivariate profiling of metabolites identified from the 2nd stage tree classification model, unsupervised principal component analysis (PCA) was conducted (Fig. 1 [4]).

The CARDIA study was approved by the Institutional Review Boards at each center, and all participants provided written informed consent. The CARDIA Publications & Presentations Committee approved this secondary analysis and waived additional consent.

## Results

### Cognitive function at year 30

A composite cognitive score comprises z-scores from cognitive function tests (Haight et al., 2018). The summation score of the four standardized cognitive tests (RAVLT, DSST, reversed Stroop, and MoCA) was generated as the adjusted composite cognitive score for the analyses at Year 30. The mean (SD) of the cognitive measurements for the DSST was 69 (16.9) with the range of 17 to 112; the Stroop was 22.3 (11.6) with the range of 0 to 107; the RAVLT was 9.3 (2.0) with the range of 2.8 to 14 at Year 30. The mean (SD) adjusted composite cognition score at Y30 was X 0.02 (3.2) with a range of −14.6 to 6.7. Cognitive status was defined using a derived composite score rather than clinical adjudication, with negative scores indicating below-normal cognitive function or mild cognitive impairment (Table 1) (Langbaum et al., 2014; Petersen et al., 1999).

**Table 1:** Cognitive measurements at Year 30

	Total		Black		White		Male		Female		
	N	Mean (SD)	N	Mean (SD)	N	Mean (SD)	N	Mean (SD)		N	Mean (SD)
DSST score, Y30	596	69.0 (16.9)	270	62.3 (16.4)	326	74.6 (15.2)	267	64.4 (16.5)		329	72.8 (16.3)
STROOP score, Y30	589	22.3 (11.6)	266	27.2 (11.9)	323	18.3 (9.5)	263	22.7 (12.7)		326	22.0 (10.6)
RAVLT score, Y30^†^	596	9.3 (2.0)	270	8.3 (1.7)	326	10.0 (1.8)	267	8.8 (1.9)		329	9.7 (1.9)
MoCA score, Y30	595	24.4 (3.6)	270	22.5 (3.7)	325	25.9 (2.8)	267	24.2 (3.7)		328	24.5 (3.6)
Composite cognitive score, Y30^‡^	589	0.02 (2.4)	266	− 1.3 (2.2)	323	1.1 (2.1)	263	− 0.6 (2.5)		326	0.5 (2.3)
Adjusted composite cognitive score, Y30^§^	588	0.02 (3.2)	266	− 1.8 (2.9)	322	1.5 (2.6)	263	− 0.6 (3.2)		325	0.5 (3.0)

^†^ RAVLT is an average of words recalled in the immediate, short, and long

^§^ z-scores from all four tests were generated (with the reverse-Stroop score)

^‡^ z-scores from the three tests (without MoCA) were generated

### Metabolites associated with cardiovascular risk factors

Metabolic syndrome is defined as the presence of at least three of the following cardiometabolic risk factors as per the adult treatment panel III guidelines and the CARDIA Study: WC (> 88 cm for women or > 102 cm for men), triglycerides (≥ 150 mg/dL), HDL–C (< 50 mg/dL for women or < 40 mg/dL for men), either SBP (≥ 130 mmHg) or DBP (≥ 85 mmHg), and fasting glucose (≥ 110 mg/dL) (NCEP Expert Panel, 2002). A total of 163 participants (26.9%) met the metabolic syndrome criteria (Black: N = 104, 37.7%; White: N = 59, 17.9%; male: N = 77, 28.2%; female: N = 86, 25.8%; *p* < 0.001, Fisher’s exact), representing the full cohort. For the PCA biplots (Fig. 2 and Supplementary Fig. 1), only participants with complete metabolite measurements were included. Significant associations between various metabolites and known risk factors for CVD and metabolic syndrome were identified. NMR spectroscopy detected metabolites such as valine, glycine, and citric acid, which showed larger component values in the non-metabolic syndrome group and smaller values in the metabolic syndrome group, whereas succinic acid exhibited greater negative values (Fig. 2). These patterns may reflect alterations in amino acid and energy metabolism associated with metabolic dysfunction. PCA shows positive correlations between citric acid, creatinine, valine, and N,N-Dimethylglycine in Supplementary Fig. 2a. Measured by the targeted LC–MS assay for amino acids, phenylalanine, tryptophan, methionine, and glutamic acid metabolites were positively correlated with the metabolic syndrome-free group. In contrast, 24 amino acids showed large negative values in the metabolic syndrome group (Supplementary Fig. 1a, b). The tryptophan pathway metabolites showed positive correlations in the non-metabolic syndrome group but large negative values in the metabolic syndrome group. These findings align with previously reported associations in the literature regarding metabolic syndrome (Supplementary Fig. 1c, d).

### Metabolites associated with cognitive performance

Five urinary metabolites (alanine, glycine, valine, sarcosine, and methionine) were measured using NMR and LC–MS. Methionine was excluded owing to non-quantified values in NMR, and sarcosine lacked sufficient data for reliable analysis. Among the normalization methods, 1D NMR-based PQN provided the best overall cross-platform concordance and was therefore used for subsequent analysis.

Associations with the adjusted composite cognitive score at Year 30 were examined to the NMR- and LC–MS-based urinary metabolites normalized using PQN, after adjusting for significant exposures. The standardized scales were applied to the metabolites and brain MRI-derived parameters in the models. The measurements of these metabolites in the two assays should show good concordance (Supplementary Table 3), supporting the robustness of our approach and mitigating the potential confounding effects associated with using multiple assays.

Table 2 presents only the significant paths for the adjusted composite cognitive score on cardiometabolic risk factors in the total multivariate linear SEM. The significant NMR-detected metabolites in the total multivariate model included valine, which showed a positive association with WC, and glycine, which showed a negative association with fasting glucose (*p* < 0.05, Table 2 [1]). Alanine also displayed a positive association with fasting insulin in the model (Supplementary Fig. 3).

**Table 2:** Summary tables of significant metabolites assayed by NMR spectroscopy and LC–MS on cardiometabolic risk factors (SBP, fasting glucose, WC, and 10-year Framingham risk) for adjusted composite cognition in the SEM

	Urinary metabolites	Cardio-vascular risk	Coefficient	$P>\left|z\right|$	Normal-based [95% CIs]	
[1] Metabolites by NMR spectroscopy†	Valine *←*	WC	0.012	0.001	0.005	0.019
	Glycine *←*	Fasting glucose	− 0.007	< 0.001	− 0.010	− 0.004
[2] Amino acids assayed by LC–MS‡	Methionine *←*	SBP	0.005	0.016	0.001	0.009
	Glycine *←*	Fasting glucose	− 0.004	< 0.001	− 0.006	− 0.002
	Isoleucine *←*	Fasting glucose	0.007	< 0.001	0.004	0.010
	Leucine *←*	Fasting glucose	0.010	< 0.001	0.006	0.013
	Phenylalanine *←*	Fasting glucose	0.003	0.020	0.001	0.006
	Valine *←*	Fasting glucose	0.011	< 0.001	0.008	0.014
	Aminoadipic acid *←*	Fasting glucose	0.006	0.011	0.001	0.010
	Serine *←*	WC	− 0.012	< 0.001	− 0.016	− 0.009
[3] Tryptophan assayed by LC–MS§	Indole-3-acetic acid *←*	Fasting glucose	− 0.007	< 0.001	− 0.011	− 0.003
	Tryptophan *←*	Fasting glucose	0.006	0.015	0.001	0.011
	Kynurenine *←*	Fasting glucose	0.007	0.001	0.003	0.011

Estimated path coefficients are represented by straight arrows.

^†^ Age, race, sex, education up to Year 30, HBP medication, and family income were significant for cognition (N = 346) (Supplementary Fig. 3).

**Fig. 3:**
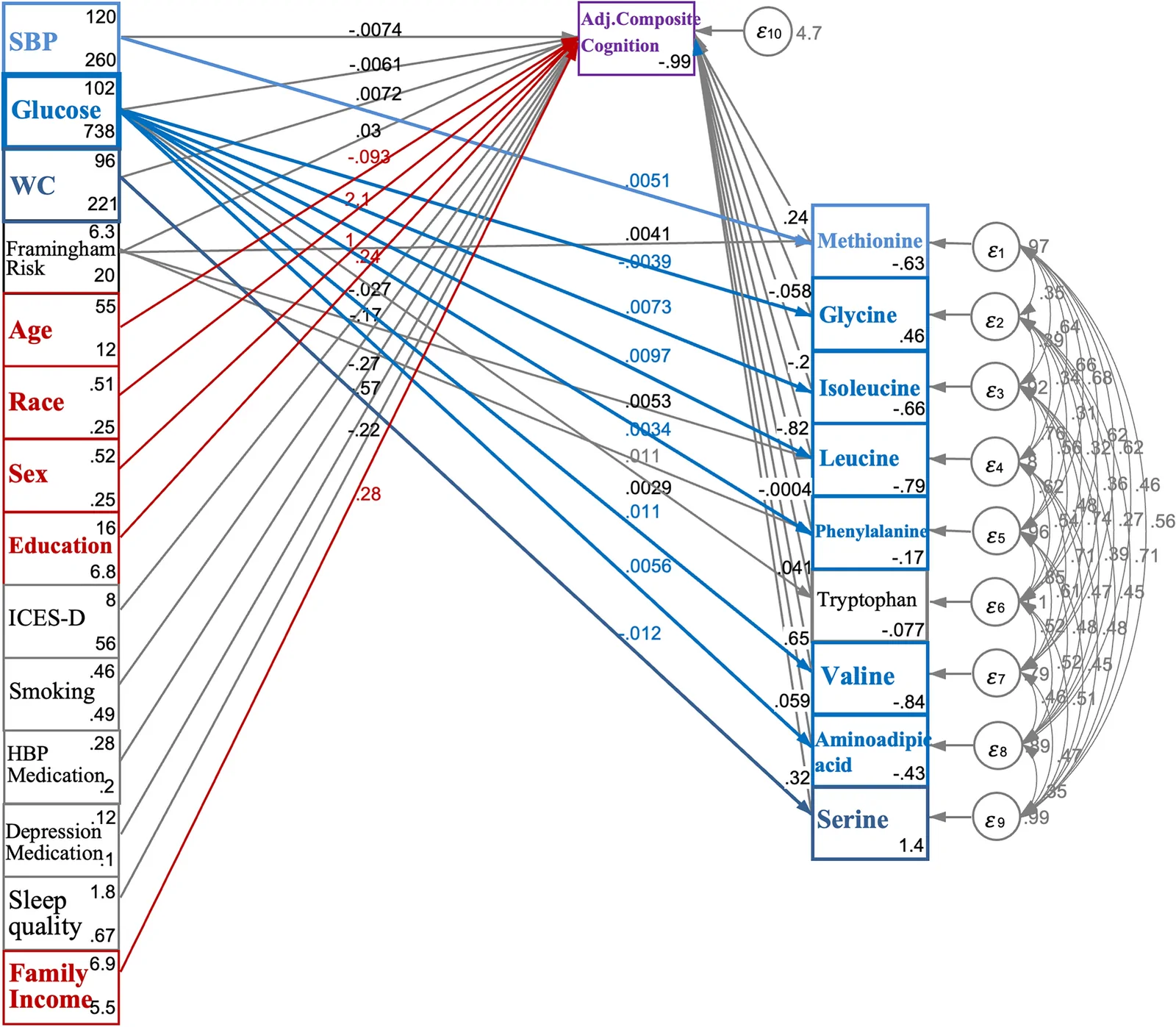
Total multivariate regression path diagram for adjusted composite cognitive score in relation to cardiometabolic risk factors (SBP, Glucose, WC, and Framingham risk) with amino acids measured by LC–MS (N = 420). The variables inside the rectangles represent the observed variables in the path diagram. The numbers inside indicate their means and variances, e.g., 120 and 260 represent the mean and variance of SBP, respectively. Paths denote direct relationships between variables, and the estimated path coefficients are indicated by straight arrows. Curved arrows indicate that error terms covary; the red color risk factors or exposures indicate those were statistically significant (*p* < 0.05) in the total model, while those in blue are significantly associated with an intermediate variable. Gray indicates significant variables in each cardiometabolic model but no longer significant in the final models. They were adjusted in the final non-recursive structural equation model

^‡^ Age, race, sex, education up to Year 30, and family income were significant for cognition (N = 420) (Fig. 3).

^§^ Age, race, sex, education up to Year 30, CES-D (center for epidemiologic studies-depression), and family income were significant for cognition (N = 413) (Supplementary Fig. 4).

For amino acids assayed by LC–MS, methionine exhibited a positive association with the mean SBP, glycine showed a negative association with fasting glucose, and isoleucine, leucine, phenylalanine, valine, and amino-adipic acid showed positive associations with fasting glucose. Serine showed a negative association with WC. In this total path diagram for amino acids, age, race, sex, education, and family income were significant (*p* < 0.05) regarding the adjusted composite cognitive score. Sex or race (adjusted for education) acted as confounding factors in the association between fasting glucose and cognition (Table 2 [2]; Fig. 3).

For the tryptophan metabolites assayed by LC–MS, indole-3-acetic acid, tryptophan, and kynurenine all showed negative associations with fasting glucose (Table 2 [3]; Supplementary Fig. 4).

### Path diagram for metabolites and brain MRI-derived parameters

**Fig. 4:**
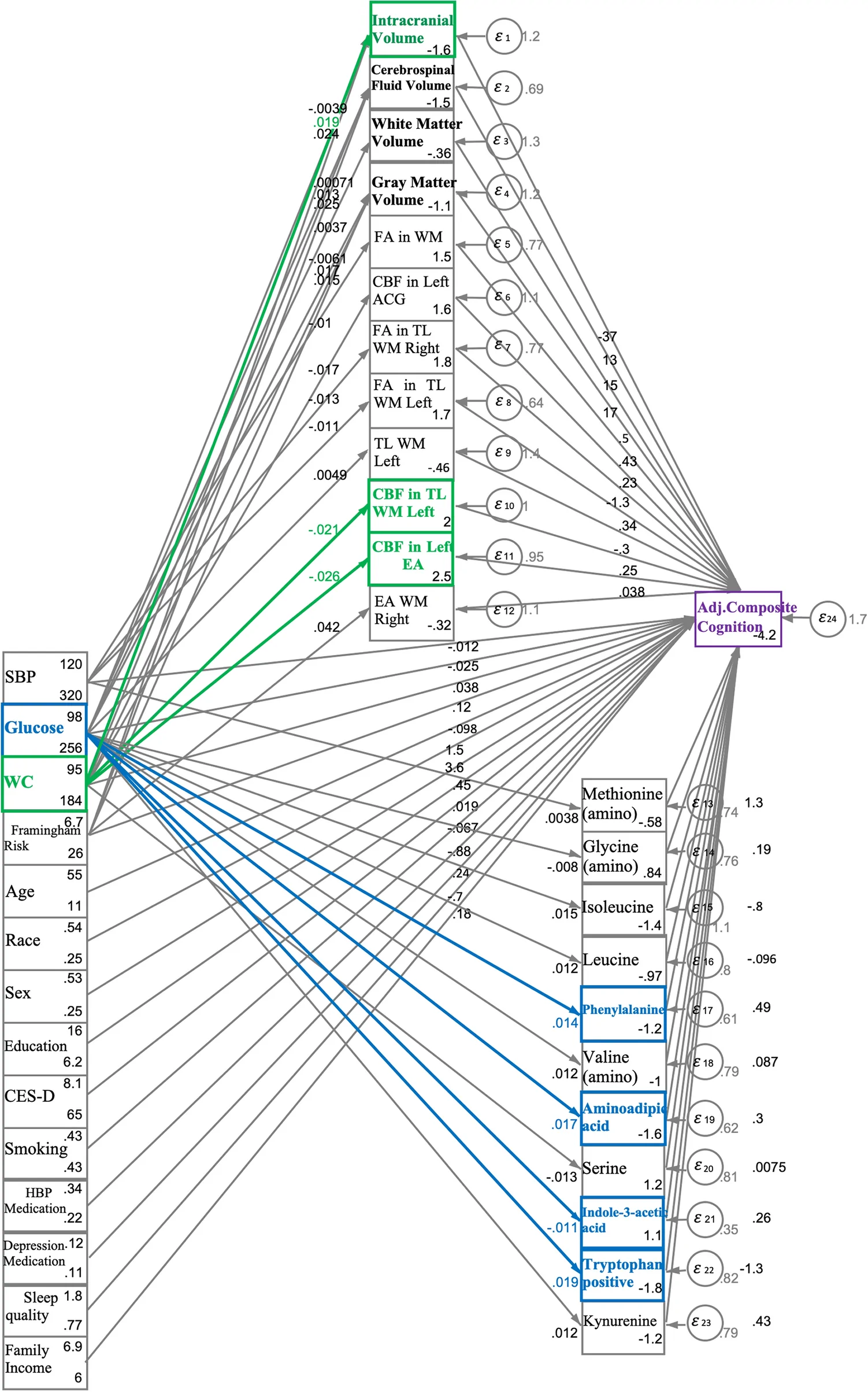
The total multivariate regression path diagram includes significant metabolites, brain MRI-derived parameters, and exposures at Year 30 (N = 74). Blue and green variables indicate those significantly associated with an intermediate variable, while gray variables were significant in each cardiometabolic model but not in the final model

Significant associations in the urinary metabolites model and brain MRI-derived parameters (Table 2 and Supplementary Table 4) from each pathway model were simultaneously included in the total path diagram (Fig. 3). The advantage of using SEM is that the indirect and total effects can be estimated in a single model, and a simple indirect pathway model can be inserted into a larger model, even using latent variables, to measure any component of the pathway model (Fig. 3) (Krull & MacKinnon, 2001; Preacher et al., 2010) (See Fig. 4).

Phenylalanine, aminoadipic acid, and tryptophan each exhibited significant **positive** associations with fasting glucose, indicating a link to hyperglycemia. In contrast, the fasting glucose itself exhibited a **negative** association with the cognitive score in the cohort (N = 74). Indole-3-acetic acid was negatively associated with fasting glucose, which is consistent with the outcomes before brain MRI-derived parameters were incorporated into the model. Furthermore, cerebral blood flow (CBF) in the temporal lobe WM left and CBF in the left entorhinal area showed significant **negative** associations with WC, indicating a connection to abdominal obesity (N = 74). The temporal lobes and entorhinal area serve as early markers of Alzheimer’s disease.

#### Profiling using metabolites and brain MRI-derived parameters

**Fig. 5:**
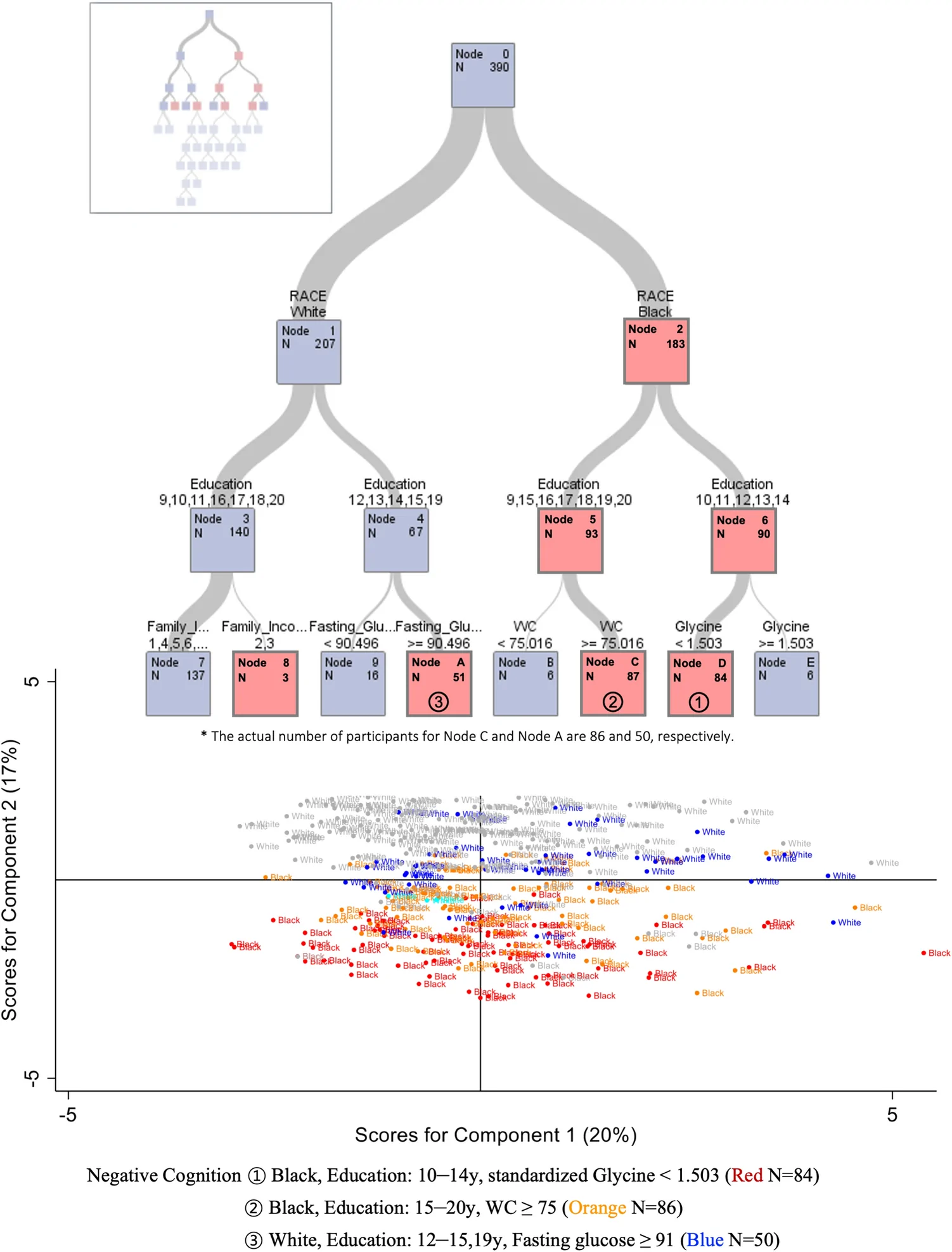
The 2.^nd^ stage classification tree for the adjusted cognitive score on the important exposures and metabolites at Year 30 (N = 390) displays node colors: red for cognitively impaired and blue for not impaired. The figure below is its PCA score plot for component 1 (20%) and component 2 (17%) with colors indicating cognitively impaired and those not impaired in gray. There were 20 participants who were cognitively impaired (i.e., z-score < 0) but were predicted as not impaired (i.e., a false negative)

Tree-based classification models and PCA were used to investigate the cardiometabolic risk-specific metabolic phenotypes (Fig. 5). Among the 390 subjects, 365 were used for PCA; the five components explained 70% of the variance across the 11 variables (age, race, education, family income, CES-D, WC, fasting glucose, glycine, tryptophan, kynurenine, and methionine). The standardized methionine concentration was significantly higher in the Black participants (mean, 0.25, N = 183) than in the White participants (mean, − 0.23, N = 207); *p* < 0.001, two-sample Wilcoxon rank-sum test (i.e., equality tests on unmatched data) (Supplementary Fig. 5).

## Discussion

Midlife obesity is associated with increased dementia risk. Small effect sizes have been reported in the literature for CVD-related cognitive impairment or dementia, even after adjusting for lifestyle, sociodemographic, and clinical exposures. In this study, we observed complex relationships between cardiometabolic risk factors, particularly obesity, and cognitive changes. Urinary metabolites and brain MRI-derived parameters were identified as potential intermediate variables in this relationship. Metabolites associated with glucose metabolism, lipids, blood pressure, and neuroinflammatory pathways (e.g., tryptophan metabolism) may serve as early biomarkers for cognitive impairment. Our findings suggest that metabolomic and neuroimaging profiling could aid in the prediction of cognitive impairment and inform potential intervention strategies.

Evidence of CVD-related cognitive impairment has been reported in the literature, with risk factors such as hypertension, hyperglycemia, obesity, and other exposures influencing cognitive impairment (Haring et al., 2013; Kivipelto et al., 2002; Ross et al., 1999). Research findings underscore the significant role of phenylalanine in modulating insulin receptor function and glucose metabolism, offering insights into the mechanisms underlying hyperglycemia and T2D progression (Zhou et al., 2022). Studies collectively highlight the association between elevated α-aminoadipic acid levels and impaired glucose metabolism, suggesting that α-aminoadipic acid could serve as a biomarker for diabetes risk and a potential modulator of glucose homeostasis (Desine, et al., 2023; Lee et al., 2019). Tryptophan is a crucial amino acid that must be obtained through the diet (Kapalka & Kapalka, 2010); its transport across the blood–brain barrier is a key regulatory mechanism, as highlighted by Palego et al. (2016) (Ovalle & P.C.N. et al., 2020). Tryptophan metabolism reflects both host metabolic processes and gut microbial activity, and these findings should be interpreted cautiously. Insulin and neutral amino acids, such as valine, leucine, isoleucine, tyrosine, and phenylalanine, play important roles in modulating this transport (Litwack & Litwack, 2018; Pardridge, 1998). Indole-3-acetic acid is associated with an increased risk of impaired cognitive function in patients undergoing hemodialysis (Lin et al., 2019). Furthermore, a 2-year pilot study in 27 adults with chronic kidney disease found that indole-3-acetic acid was correlated with anxiety (ρ = 0.52, p = 0.005) and depression (ρ = 0.39, p < 0.05) (Karu et al., 2016). Glycine is an amino acid used by the body to synthesize proteins and has been shown to reduce daytime sleepiness and improves cognition (Glenn et al., 2019; Inagawa et al., 2006). Gannon et al. (2002) showed that oral glycine stimulates the secretion of gut hormones, enhancing insulin action and lowering blood glucose levels in humans. Glycine-induced insulin secretion can benefit diabetes management (Yan-Do et al., 2016), and insulin dysfunction, as occurs in type 2 diabetes, is linked to Alzheimer-related brain pathology (Arvanitakis et al., 2004; Ott et al., 1996). The relationship between urinary and circulating metabolites is not necessarily concordant, as urinary metabolites reflect excretion rather than systemic levels (Bouatra et al., 2013). While lower circulating glycine is linked to higher cardiometabolic risk (Wang et al., 2011), higher urinary glycine may reflect increased excretion or altered metabolism. Higher methionine intake may be associated with an increased risk of cognitive decline and MCI in humans, potentially via oxidative stress and alterations in methylation pathways (Kinoshita et al., 2021; Xi et al., 2023). Elevated kynurenine levels were observed in individuals with major depression (Myint et al., 2011; Sublette et al., 2011). Myint et al. (2007) suggested that an imbalance between neuroprotective and neurotoxic kynurenine metabolites can contribute to the pathophysiology of depression. This disrupted kynurenine metabolism has also been implicated in interferon-induced depression and other neuropsychiatric side effects (Myint et al., 2011).

A previous study investigated the ventromedial part of the temporal lobes and the entorhinal cortex as early AD markers (Hoesen et al., 1991). In this study, total ICV acted as an intermediate variable in the **positive** association with WC related to abdominal obesity. Measures of brain physiology, including CBF in the left temporal lobe WM and CBF in the left entorhinal area, acted as intermediate variables in the **negative** associations with WC. This finding aligns with the review that obesity, a risk factor for VaD, not only reduces blood supply to the brain but also increases fat cells (adipocytes) that damage brain WM, resulting in cognitive and intellectual capacity loss (Anjum et al., 2018).

Evidence from Whitmer’s review (2007) suggests a link between obesity and increased dementia risk, possibly owing to higher adipokine levels associated with obesity, linked to decreased WM in the brain (Arnoldussen et al., 2014; Kiliaan et al., 2014). These results support the existing evidence linking obesity and metabolic dysfunction to reduced brain health. Monitoring midlife obesity-related indicators (such as WC) and brain structure/function could enhance early dementia prevention efforts. Our findings indicate that higher fasting glucose and abdominal obesity were associated with poorer cognitive outcomes and reduced brain perfusion in Alzheimer’s-vulnerable regions. Metabolites, such as phenylalanine, α-aminoadipic acid, and tryptophan, were linked to hyperglycemia and cognitive decline. Adverse outcomes were also associated with older age, lower education and income, depressive symptoms, and smoking. Brain MRI markers, particularly reduced FA and voxel activation in the frontal and temporal regions, were associated with elevated cardiometabolic risk. These patterns were more pronounced among Black participants.

### Strengths and limitations

This study employed a fusion analytic method to explore relationships in an epidemiological settting. The applied biostatistical approach help identify complex epidemiologic and phenotypic relationships across cardiovascular and neurological disease networks. While primarily a statistical contribution, the findings are informed by biomedical expertise, integrating brain MRI and metabolomic data to identify potential physiological markers of cognitive impairment and metabolic dysfunction. One strength of the study is the use of SEM, which allows the estimation of indirect and total effects from a single model. It permits embedding a simple pathway model into a larger model to measure any component of the indirect association (Fig. 1 [1–3] (Krull & MacKinnon, 2001; Preacher et al., 2010). The study included adjustments for lifestyle, sociodemographic, and clinical exposures that may influence the exposures and outcomes. A systematic, multiple-analytic process was used to ensure robustness of the findings. The test hypothesis is only one for each association in the univariate regression. Bootstrapped standard errors, confidence intervals, and the Swain correction were applied to obtain conservative significance estimates. A multi-stage examination approach using various analytical methods was employed to validate the outcomes. Several other significant exposures were adjusted applying robust analytical methods. Nevertheless, findings should be interpreted carefully and supported by further follow-up data.

Direct pathways between amino acids and cognition are not well established. Future investigations could further explore specific relationships, such as those within metabolic pathways involving tryptophan and/or kynurenine, in relation to the gut-brain axis and metabolites potentially associated with brain inflammation. Other biological pathways underlying the associations between obesity and cognitive impairment may involve gut-brain axis and central inflammation processes (Solas et al., 2017). Tryptophan catabolites may contribute to immune responses and neurodegenerative processes (Gasaly et al., 2021; Hebbrecht et al., 2021). Pro-inflammatory metabolites and immune-regulatory dysfunction have been implicated in gut dysbiosis and cognitive decline (Solas et al., 2017). The gut microbiota may link high-fat or otherwise unbalanced diets to impaired cognition, known as the “gut-brain axis” (Solas et al., 2017). Studies suggest that elevated BMI in midlife significantly increases dementia risk, potentially attributed to heightened inflammation and elevated cytokine and hormone levels produced by adipose tissues, which are recognized risk factors for AD (Arnoldussen et al., 2014; Emmerzaal et al., 2014; Hata et al., 2019; Yaffe et al., 2004). Continued follow-up of the cohort will be a valuable source of information for developing a cognitive impairment risk assessment tool with 5-year follow-up intervals. Insights gained from metabolomic analysis could improve risk prediction for stratified cohorts relevant to cognitive outcomes. Given the relatively small sample size, generalizability may be limited, and external validation in independent cohorts is warranted.

## Conclusion

Urinary metabolites and brain imaging markers may be key to understanding and predicting the cognitive trajectory of individuals with cardiometabolic risk, including hyperglycemia and obesity. Prognostic factors identified in this study may inform risk assessment tools for cognitive impairment. Current clinical practice often overlooks specific metabolic, genetic, or clinical markers, assuming they are of minor importance in managing CVD-related risk or cognitive outcomes. This study demonstrates the potential application of these newly identified prognostic markers in the management and risk stratification of cognitive impairment in adults. Such advancements have the potential to improve long-term prediction of cognitive impairment and enhance overall brain health in the general population.

## Supplementary Information

Below is the link to the electronic supplementary material.

Supplementary file1 (DOCX 607 KB)

## Data Availability

The data supporting this study are not publicly available due to ethical and legal restrictions under CARDIA study policies.
