# Supplementary material for: Metabolic pathways associated with cardiometabolic risk effects on cognition in middle-aged adults: the CARDIA study
Source: Metabolomics. 2026 Jun 17;22(4):99. doi: 10.1007/s11306-026-02458-w (PMC13275628; doi:10.1007/s11306-026-02458-w)
Supplement: Supplementary file 1 — Supplementary file1 (DOCX 607 KB) [file 11306_2026_2458_MOESM1_ESM.docx]

#### **Supplementary**

**Supplementary Table 1** Cardio-metabolic risk factors at Year 30 (males and females by race)

|  |  | Total | Mean (SD) | Black Male | Mean (SD) | White Male | Mean (SD) | Black Female | Mean (SD) | White Female | Mean (SD) |
| --- | --- | --- | --- | --- | --- | --- | --- | --- | --- | --- | --- |
| Hypertension | SBP **††** | 606 | 119.3 (16.1) | 118 | 123.8 (13.5) | 155 | 118.4 (13) | 158 | 125.4 (19.1) | 175 | 111.6 (13.8) |
|  | DBP **††** | 606 | 72.8 (11.4) | 118 | 76.2 (10.3) | 155 | 72.5 (9.3) | 158 | 76.9 (11.1) | 175 | 67.1 (11.6) |
| Dyslipidaemia | HDL–C | 601 | 60.2 (20.5) | 116 | 52.5 (14) | 155 | 49.2 (11.9) | 155 | 67.1 (26.1) | 175 | 68.9 (17.9) |
|  | LDL–C | 593 | 110.6 (32.7) | 114 | 106.3 (35.1) | 151 | 113.6 (29.9) | 154 | 109.9 (35.1) | 174 | 111.5 (31) |
|  | Triglycerides (mg/dL) | 599* | 105.8 (66.7) | 115 | 106.1 (64.8) | 154 | 127.7 (76.9) | 155 | 98.5 (64.7) | 175 | 92.8 (54.6) |
| Hyperglycaemia | Fasting glucose (mg/dL) | 601 | 100.5 (25.2) | 116 | 107.7 (39.2) | 155 | 102.7 (22.4) | 155 | 99.5 (22.3) | 175 | 94.7 (15) |
|  | HOMA-IR **††** | 593 | 3.1  (2.8) | 112 | 3.4 (2.7) | 153 | 3.5 (3.5) | 154 | 3.4 (2.8) | 174 | 2.2 (1.9) |
|  | eGFR **††** | 596 | 91.3 (15.5) | 113 | 92 (19) | 153 | 87.6 (11.1) | 155 | 96.1 (18.1) | 175 | 89.9 (12.6) |
|  | Fasting insulin **††** | 598 | 11.9  (8.7) | 115 | 12.8 (9) | 155 | 12.8 (9) | 154 | 13.5 (9.6) | 174 | 9.1 (6.6) |
| (Abdominal) Obesity | BMI **††** | 605 | 29.4  (6.2) | 117 | 30.3 (5.8) | 155 | 29.1 (4.6) | 158 | 32.2 (6.9) | 175 | 26.6 (5.8) |
|  | WC **††** | 605 | 94.6 (15.2) | 118 | 100.8 (14.7) | 155 | 99.8 (12.6) | 158 | 95 (14.1) | 174 | 85.2 (14) |
| CVD | 10-year  Framingham risk, male | 261 | 7.7 (4.7) | 112 | 7.8 (5.4) | 149 | 7.7 (4.1) |  |  |  |  |
|  | 10-year  Framingham risk, female **††** | 323 | 4.7 (3.7) |  |  |  |  | 153 | 5.8 (4.5) | 170 | 3.7 (2.4) |
| * Omitted two distinctive observations of triglycerides > 600 (2366 and 3234 mg/dL)  **††** *p* < 0.001 for females by race | | | | | | | | | | | |

**Supplementary Table 2** Significant exposures, confounding factors, and tryptophan mediators by LC–MS in the structural equation models by each cardio-metabolic factor

|  |  | Hypertension | | Hyperglycaemia | | | (Abdominal) Obesity | | CVD |
| --- | --- | --- | --- | --- | --- | --- | --- | --- | --- |
|  |  | SBP | DBP | Fasting glucose | HOMA-IR | Fasting insulin | BMI | WC | 10-Year Framingham Risk |
| Significant exposures | Race |  |  |  | ⚫ | ⚫ |  | ⚫ | ⚫ |
|  | Sex | ⚫ | ⚫ |  | ⚫ | ⚫ | ⚫ |  |  |
|  | Age |  |  | ⚫ | ⚫ | ⚫ | ⚫ | ⚫ |  |
|  | Education | ⚫ | ⚫ | ⚫ | ⚫ | ⚫ | ⚫ | ⚫ | ⚫ |
|  | CES–D |  |  |  | ⚫ | ⚫ |  |  |  |
|  | Smoking |  |  |  |  |  | ⚫ |  |  |
|  | HBP medication | ⚫ | ⚫ |  |  |  |  |  |  |
|  | Depression medication |  |  |  | ⚫ | ⚫ | ⚫ |  |  |
|  | Sleep quality | ⚫ | ⚫ |  |  |  | ⚫ |  |  |
|  | Family Income | ⚫ | ⚫ | ⚫ | ⚫ | ⚫ | ⚫ | ⚫ | ⚫ |
| Confounding factors | Conf Race | ⚫ | ⚫ | ⚫ |  |  | ⚫ |  |  |
|  | Conf Sex |  |  | ⚫ |  |  |  | ⚫ | ⚫ |
|  | Conf Education |  |  |  |  |  |  |  |  |
|  | Conf Age |  |  |  |  |  |  |  | ⚫ |
| Significant metabolites | Alanine (amino acids) | ⚫ | ⚫ | ⚫ | ⚫ | ⚫ | ⚫ | ⚫ | ⚫ |
|  | Asparagine |  |  |  |  |  |  |  |  |
|  | Ethanolamine |  |  | ⚫ |  | ⚫ | ⚫ | ⚫ |  |
|  | Glutamic acid | ⚫ |  |  |  |  |  |  | ⚫ |
|  | Glutamine |  |  | ⚫ |  |  |  |  |  |
|  | Glycine (amino acids) |  |  | ⚫ |  |  |  | ⚫ | ⚫ |
|  | Histidine |  |  |  |  |  |  | ⚫ |  |
|  | Isoleucine | ⚫ | ⚫ | ⚫ | ⚫ | ⚫ | ⚫ | ⚫ | ⚫ |
|  | Leucine |  | ⚫ | ⚫ | ⚫ | ⚫ | ⚫ | ⚫ | ⚫ |
|  | Lysine244 |  |  |  |  |  |  |  |  |
|  | Methionine (amino acids) | ⚫ |  | ⚫ | ⚫ | ⚫ | ⚫ | ⚫ | ⚫ |
|  | Phenylalanine | ⚫ | ⚫ | ⚫ | ⚫ | ⚫ | ⚫ | ⚫ | ⚫ |
|  | Serine |  |  | ⚫ | ⚫ | ⚫ |  | ⚫ |  |
|  | Threonine | ⚫ | ⚫ |  |  |  |  |  |  |
|  | Tryptophan | ⚫ |  | ⚫ | ⚫ | ⚫ | ⚫ | ⚫ | ⚫ |
|  | Tyrosine | ⚫ | ⚫ | ⚫ | ⚫ | ⚫ | ⚫ | ⚫ | ⚫ |
|  | Valine (amino acids) | ⚫ | ⚫ | ⚫ | ⚫ | ⚫ | ⚫ | ⚫ | ⚫ |
|  | Beta-aminoisobutyric acid |  |  |  |  |  |  |  |  |
|  | Cystathionine282 |  |  |  |  |  |  |  |  |
|  | methylhistidine3 |  |  |  |  |  | ⚫ |  |  |
|  | 1-Methylhistidine |  | ⚫ |  |  |  |  | ⚫ |  |
|  | Hydroxylysine | ⚫ | ⚫ |  | ⚫ | ⚫ | ⚫ | ⚫ | ⚫ |
|  | Aminoadipic acid |  |  | ⚫ | ⚫ | ⚫ |  | ⚫ | ⚫ |
|  | Beta-alanine |  |  |  |  |  |  |  |  |
| **Mediators** | Glycine (amino acids) |  |  | ⚫ |  |  |  |  |  |
|  | Isoleucine |  |  | ⚫ |  |  |  |  | ⚫ |
|  | Leucine |  |  | ⚫ |  |  |  |  |  |
|  | Methionine (amino acids) | ⚫ |  |  |  |  |  |  | ⚫ |
|  | Phenylalanine |  |  | ⚫ |  |  |  |  | ⚫ |
|  | Serine |  |  |  |  |  |  | ⚫ |  |
|  | Tryptophan |  |  | ⚫ |  |  |  |  |  |
|  | Threonine |  |  |  |  |  |  |  |  |
|  | Valine (amino acids) |  |  | ⚫ |  |  |  |  |  |
|  | methylhistidine3 |  |  |  |  |  |  |  |  |
|  | Hydroxylysine |  |  |  |  |  | ⚫ |  |  |
|  | Aminoadipic acid |  |  | ⚫ |  |  |  |  |  |
|  | Beta-alanine |  |  |  |  |  |  |  |  |
| eGFR (Hyperglycaemia) was not significant for Adj. composite Cognition (*p=*0.05)  HDL–C, LDL–C and Triglycerides (Dyslipidaemia) were not significant for Adj. composite Cognition (*p=*0.05) | | | | | | | | | |

**Supplementary Table 3** Spearman's rank correlation coefficient of metabolites measured by both NMR and LC–MS

|  | | Alanine (LC–MS) | | | | Glycine (LC–MS) | | | | Valine (LC–MS) | | | | Sarcosine (LC–MS) | | | |
| --- | --- | --- | --- | --- | --- | --- | --- | --- | --- | --- | --- | --- | --- | --- | --- | --- | --- |
|  |  | [1] | [2] | [3] | [4] | [1] | [2] | [3] | [4] | [1] | [2] | [3] | [4] | [1] | [2] | [3] | [4] |
| Alanine (NMR) | Corr. | 0.97 | 0.96 | 0.69 | 0.90 | 0.69 | 0.63 | 0.03 | 0.32 | 0.86 | 0.83 | 0.03 | 0.57 | 0.31 | 0.38 | -0.26 | 0.05 |
|  | N | 501 | | | | 501 | | | | 499 | | | | 95 | | | |
| Glycine (NMR) | Corr. | 0.74 | 0.68 | 0.09 | 0.40 | 0.98 | 0.97 | 0.82 | 0.96 | 0.75 | 0.69 | -0.25 | 0.34 | 0.23 | 0.25 | -0.26 | 0.08 |
|  | N | 546 | | | | 546 | | | | 544 | | | | 96 | | | |
| Valine (NMR) | Corr. | 0.74 | 0.69 | 0.16 | 0.41 | 0.57 | 0.53 | 0.02 | 0.26 | 0.89 | 0.86 | 0.24 | 0.64 | 0.30 | 0.40 | -0.38 | -0.02 |
|  | N | 402 | | | | 402 | | | | 401 |  |  |  | 90 | | | |
| Sarcosine (NMR) | Corr. | 0.55 | 0.66 | -0.24 | -0.16 | 0.40 | 0.44 | 0.11 | 0.00 | 0.55 | 0.62 | -0.10 | -0.12 | -0.36 | 0.50 | -0.10 | -0.90 |
|  | N | 23 | | | | 23 | | | | 23 | | | | 5 | | | |
| [1] The data without application of normalization (with standardization)  [2] Normalization by the PQN Creatinine method (with standardization)  [3] PQN using the 1D NMR data for NMR, and PQN using the final stage amino acids data for LC–MS (with standardization)  [4] PQN, using the 1D NMR data for both NMR and LC–MS (with standardization) | | | | | | | | | | | | | | | | | |

Alanine, glycine, valine, sarcosine and methionine were measured by both NMR and LC–MS. Alanine, glycine, and valine showed relatively good concordance across assays both prior to normalization [1] and after PQN creatinine normalization [2]. Methionine was excluded from correlation analyses due to being non-quantified in NMR, and sarcosine had few data points to allow reliable correlation analysis. Valine exhibited 33.3% missing values by NMR compared with 0.5% by LC–MS, which likely contributed to a reduced correlation after PQN normalization (ρ from 0.90 to 0.64) [4]. Glycine exhibited 9.3% missing values by NMR versus 0.17% by LC-MS. Although the proportion of missing data likely influenced PQN performance, the 1D NMR-based PQN method still yielded stronger overall cross-platform concordance.

**Supplementary Table 4** Significant brain MRI-derived parameters in relation to cardio-metabolic risk factors (SBP, fasting glucose, WC, 10-Year Framingham Risk) for adjusted composite cognition* in the SEM (N=155)

| Brain MRI-derived parameters | Cardio-vascular Risk | Coefficient | $P>\left\vert z \right\vert$ | Normal-based [95% CIs] | |
| --- | --- | --- | --- | --- | --- |
| Fractional Anisotropy in White Matter$\leftarrow$ | SBP | -0.014 | 0.007 | -0.024 | -0.004 |
| Fractional Anisotropy in Temporal Lobe White Matter Right$\leftarrow$ | SBP | -0.017 | 0.003 | -0.028 | -0.006 |
| Fractional Anisotropy in Temporal Lobe White Matter Left$\leftarrow$ | SBP | -0.014 | 0.006 | -0.024 | -0.004 |
| Total White Matter Volume$\leftarrow$ | Fasting glucose | 0.015 | 0.023 | 0.002 | 0.028 |
| Total Temporal Lobe White Matter Left $\leftarrow$ | Fasting glucose | 0.016 | 0.024 | 0.002 | 0.029 |
| Cerebral Blood Flow in Left Anterior Cingulate Gyrus$\leftarrow$ | WC | -0.013 | 0.016 | -0.024 | -0.002 |
| Cerebral Blood Flow in Temporal Lobe White Matter Left$\leftarrow$ | WC | -0.014 | 0.027 | -0.027 | -0.002 |
| Cerebral Blood Flow in Left Entorhinal Area $\leftarrow$ | WC | -0.015 | 0.012 | -0.026 | -0.003 |
| Total Right Entorhinal Area Volume$\leftarrow$ | 10-Year Framingham Risk | 0.067 | 0.005 | 0.021 | 0.114 |
| Only significant paths from the total multivariate linear SEM are listed  Race, sex, and education up to Year 30 were significant regarding cognition  * Sum of z-scores from RAVLT, DSST, reversed Stroop, and MoCA | | | | | |

Total volumes of the brain, WM and GM, cerebrospinal fluid, and (normalised) mean factional anisotropy (FA) in the right MFC were considered statistically and clinically significant parameters. Total cerebrospinal fluid and total GM volume were also included in the total brain MRI model with the urinary metabolites, even though they were not individually significant in the total brain MRI model (**Fig. 1** [1–3]).

(a) (b)

**Supplementary Fig. 1**

The biplot for (a) no metabolic syndrome and (b) metabolic syndrome groups (N=184 vs 81)
using principal component analysis of PQN-normalized amino acids;

The biplot for (c) no metabolic syndrome and (d) metabolic syndrome groups (N=85 vs 31)
using principal component analysis of PQN-normalized tryptophan pathway metabolites.

(c) (d)

(a)

(b)

(c)

**Supplementary Fig. 2**

(a) PCA of the probabilistic quotient normalization (PQN) applied to NMR spectra data (10 metabolites with <50% missing cases);

(b) PCA of the probabilistic quotient normalization (PQN) applied to amino acids (24 metabolites with <50% missing cases);

(c) PCA of the probabilistic quotient normalization (PQN) applied to tryptophan (11 metabolites with <50% missing cases).

6.8

16

251

119

246

96

843

102

20

6.2

56

7.9

.25

.56

**Glycine**

.76

.77

**Valine**

-1.6

1

12

55

.46

.43

-2.5

4.6

.1

.12

.63

1.8

5.2

7

.18

.23

.25

.55

Citric acid

.75

.97

-.0071

.0042

.012

.24

.29

-.0061

.011

-.0082

.034

-.038

2.1

.23

-.061

-.084

.019

-.46

-.15

.27

-.61

.96

.099

-.0048

.33

.21

**Education**

SBP

**WC**

**Glucose**

Framingham
Risk

CES–D

**Race**

**Age**

Smoking

Depression
Medication

Sleep
quality

**Family
Income**

**HBP
Medication**

**Sex**

**Adj.Composite**
**Cognition**

1

2

3

$$\varepsilon$$

$$\varepsilon$$

$$\varepsilon$$

4

$$\varepsilon$$

**Supplementary Fig. 3** Total multivariate regression path diagram for the adjusted composite cognitive score on cardio-metabolic risk factors (SBP, fasting glucose, WC, and Framingham risk) with NMR metabolites (N=346). The variables inside the rectangles are observed variables in this path diagram. The figures inside the rectangles are mean and variance of the variables. For instance, 119 and 251 indicate mean and variance of SBP, respectively. Paths are direct relationships between variables. Estimated path coefficients are represented by straight arrows. Curved arrows specify that error terms covary.

In the total path diagram of the metabolites measured by NMR model which included (1) WC (instead of BMI), and (2) fasting glucose (instead of fasting insulin), age, race, sex, education (up to Year 30), HBP medication and family income were significant (*p* < 0.05) regarding the adjusted composite cognitive score. Fasting glucose was significant regarding **glycine** (Coef. -0.007). WC was significant regarding **valine** (Coef. 0.012).

6.8

16

265

119

210

94

637

101

20

6.2

60

8

.24

.58

1.4

1

1

-.63

2

1

12

55

1

3

4.7

.74

1.9

5.4

7

.21

.3

.25

.54

-.64

4

1.1

-.0057

-.0072

.0063

.084

.21

-.0059

-.0057

-.0028

.011

-.046

2.2

.13

.053

-.11

-.47

.37

-.19

1.2

-.13

.0067

-.049

.63

SBP

**Glucose**

Education

CES–D

Sleep
quality

Family
Income

HBP
Medication

WC

Framingham
Risk

Race

Age

Sex

$$\varepsilon$$

$$\varepsilon$$

$$\varepsilon$$

$$\varepsilon$$

**Indole-3-acetic
acid**

**Tryptophan**

**Kynurenine**

**Adj.Composite**
**Cognition**

**Supplementary Fig. 4** Total multivariate regression path diagram for the adjusted composite cognitive score on cardio-metabolic risk factors (SBP, fasting glucose, WC, and Framingham risk) with tryptophan (N=413)

The same analytic approaches were applied to the models for tryptophan measured by LC–MS; tryptophan pathway metabolites which are mediators on each cardio-metabolic risk factor, and exposures which are confounding factors in each association have been examined.

Individuals with higher **methionine**

**Methionine is higher in the black ethnic group**
(*p* < 0.001, two-sample Wilcoxon rank-sum test)

**Supplementary Fig. 5** The PCA score plot for the component 1 (20%) and the component 2 (17%); colour–cognitively impaired/ grey–not impaired; a darker red colour scale indicates higher methionine
